# Supplementary material for: Using Micro- and Macro-Level Network Metrics Unveils Top Communicative Gene Modules in Psoriasis
Source: Genes (Basel). 2020 Aug 10;11(8):914. doi: 10.3390/genes11080914 (PMC7464240; doi:10.3390/genes11080914)
Supplement: Supplementary file 1 [file genes-11-00914-s001.zip › supplementary_materials_version2/Table S2.pdf]

Table S2. Top 50 upregulated DEGs among lesional and non-lesional samples.

prob_id	Gene symbol	logFC	P.Value	adj.P.Val
220528_at	VNN3	7.21	1.04E-63	5.68E-60
211906_s_at	SERPINB4	6.51	2.70E-59	6.43E-56
205863_at	S100A12	6.39	6.49E-61	1.87E-57
232220_at	S100A7A	5.79	4.15E-67	3.78E-63
220655_at	TNIP3	5.53	6.57E-52	6.31E-49
205513_at	TCN1	5.15	3.29E-63	1.50E-59
207602_at	TMPRSS11D	5.06	9.80E-61	2.55E-57
220664_at	SPRR2C	4.89	5.54E-61	1.68E-57
238689_at	ADGRF1	4.75	7.00E-48	3.33E-45
204470_at	CXCL1	4.66	2.25E-42	5.89E-40
1569555_at	GDA	4.47	6.22E-57	1.17E-53
207367_at	ATP12A	4.39	1.85E-43	5.63E-41
202859_x_at	CXCL8	4.21	2.42E-41	5.51E-39
206561_s_at	AKR1B10	4.15	3.12E-63	1.50E-59
204385_at	KYNU	4.05	3.35E-69	9.15E-65
218990_s_at	SPRR3	4.05	1.91E-35	2.17E-33
220810_at	CLCA3P	3.98	8.19E-31	5.53E-29
232170_at	S100A7A	3.98	9.35E-51	6.91E-48
1553434_at	CYP4Z2P	3.94	1.67E-39	3.13E-37
219554_at	RHCG	3.92	5.65E-64	3.43E-60
203535_at	S100A9	3.92	4.55E-53	5.08E-50
209719_x_at	SERPINB3	3.91	1.37E-62	5.00E-59
205242_at	CXCL13	3.91	1.33E-43	4.14E-41
232074_at	PRSS27	3.91	7.03E-61	1.92E-57
216876_s_at	IL17A	3.90	2.02E-39	3.74E-37
217315_s_at	KLK13	3.87	5.14E-52	5.11E-49
221404_at	IL36A	3.86	1.25E-34	1.30E-32
205660_at	OASL	3.85	2.30E-67	2.51E-63
205476_at	CCL20	3.84	1.29E-37	1.92E-35
219403_s_at	HPSE	3.81	1.26E-64	8.63E-61
204475_at	MMP1	3.78	1.25E-25	4.63E-24
220249_at	HYAL4	3.78	8.92E-40	1.72E-37
239430_at	IGFL1	3.71	3.05E-49	1.75E-46
212531_at	LCN2	3.58	4.18E-50	2.72E-47
1554914_at	PLA2G4D	3.54	3.35E-68	4.58E-64
220322_at	IL36G	3.53	3.86E-63	1.62E-59
207901_at	IL12B	3.43	1.77E-33	1.62E-31
206367_at	REN	3.40	3.96E-43	1.14E-40
217110_s_at	MUC4	3.39	1.70E-26	6.97E-25
231033_at	DSC2	3.35	3.19E-39	5.82E-37

223869_at	SOST	3.34	3.52E-28	1.75E-26
204733_at	KLK6	3.33	8.40E-34	7.93E-32
227735_s_at	C10orf99	3.31	2.09E-45	7.52E-43
1558549_s_at	VNN1	3.28	7.86E-52	7.41E-49
219270_at	CHAC1	3.26	1.78E-39	3.31E-37
216258_s_at	SERPINB13	3.21	1.65E-48	8.57E-46
220745_at	IL19	3.14	1.61E-40	3.38E-38
234408_at	IL17F	3.14	7.62E-31	5.16E-29
224071_at	IL20	3.14	8.91E-31	5.99E-29
223906_s_at	TEX101	3.13	6.33E-41	1.40E-38
210164_at	GZMB	3.13	5.33E-50	3.31E-47
