# Supplementary material for: Using Micro- and Macro-Level Network Metrics Unveils Top Communicative Gene Modules in Psoriasis
Source: Genes (Basel). 2020 Aug 10;11(8):914. doi: 10.3390/genes11080914 (PMC7464240; doi:10.3390/genes11080914)
Supplement: Supplementary file 1 [file genes-11-00914-s001.zip › supplementary_materials_version2/Table S3.pdf]

Table S3. Top 50 downregulated DEGs among lesional and non-lesional samples

prob_id	Gene symbol	logFC	P.Value	adj.P.Val
228241_at	AGR3	-4.05	1.66E-27	7.63E-26
207326_at	BTC	-3.62	1.86E-36	2.41E-34
217059_at	MUC7	-3.36	2.11E-27	9.56E-26
229477_at	THRSP	-3.34	6.61E-18	9.61E-17
204712_at	WIF1	-3.18	8.52E-29	4.54E-27
206143_at	SLC26A3	-3.12	5.29E-14	4.80E-13
220196_at	MUC16	-3.11	4.96E-21	1.05E-19
204515_at	HSD3B1	-2.96	2.02E-12	1.50E-11
1553211_at	ANKFN1	-2.88	1.29E-19	2.30E-18
214240_at	GAL	-2.85	7.09E-12	4.92E-11
205813_s_at	MAT1A	-2.73	1.60E-25	5.84E-24
205843_x_at	CRAT	-2.73	2.02E-22	5.07E-21
215682_at	LOC440792	-2.72	9.52E-25	3.14E-23
237748_at	SCGB2B2	-2.68	4.42E-19	7.36E-18
208331_at	BPY2	-2.66	5.91E-07	2.19E-06
239929_at	PM20D1	-2.66	1.22E-09	6.42E-09
228403_at	ENHO	-2.62	1.61E-23	4.57E-22
216881_x_at	PRB4	-2.59	6.46E-31	4.42E-29
237076_at	NCSTN	-2.54	6.38E-29	3.47E-27
202218_s_at	FADS2	-2.50	2.05E-11	1.34E-10
219619_at	DIRAS2	-2.49	1.10E-23	3.18E-22
206393_at	TNNI2	-2.49	4.29E-22	1.04E-20
206794_at	ERBB4	-2.48	1.11E-23	3.21E-22
1557598_at	LOC101927870	-2.45	8.38E-18	1.20E-16
205979_at	SCGB2A1	-2.44	1.83E-23	5.17E-22
1560011_at	JRK	-2.42	2.64E-27	1.18E-25
205404_at	HSD11B1	-2.42	3.10E-26	1.23E-24
230045_at	CNTN2	-2.41	1.41E-29	8.22E-28

205856_at	SLC14A1	-2.41	2.70E-29	1.52E-27
234513_at	ELOVL3	-2.41	5.76E-11	3.57E-10
231963_at	ANKRD33B	-2.39	2.57E-36	3.27E-34
207430_s_at	MSMB	-2.37	1.07E-16	1.35E-15
214981_at	POSTN	-2.36	1.02E-32	8.55E-31
1563209_a_at	MACROD2	-2.32	1.46E-28	7.65E-27
1552566_at	BTBD16	-2.31	3.53E-25	1.23E-23
227123_at	RAB3B	-2.31	7.12E-35	7.56E-33
206811_at	ADCY8	-2.29	5.47E-15	5.59E-14
213706_at	GPD1	-2.28	1.10E-20	2.23E-19
1555318_at	HIF3A	-2.27	8.49E-26	3.20E-24
1558393_at	KRT7	-2.26	4.90E-17	6.42E-16
214558_at	GPR12	-2.25	1.63E-18	2.55E-17
1554920_at	SCEL	-2.22	5.35E-12	3.77E-11
229151_at	SLC14A1	-2.20	5.04E-20	9.40E-19
234980_at	TMEM56	-2.19	5.97E-17	7.74E-16
234436_x_at	OBP2A	-2.16	1.53E-13	1.32E-12
207955_at	CCL27	-2.15	4.94E-30	3.02E-28
224588_at	XIST	-2.14	0.000133	0.000354
222049_s_at	RBP4	-2.13	6.24E-13	4.97E-12
227174_at	WDR72	-2.12	2.12E-29	1.21E-27
1559296_at	ADAMTS9-AS2	-2.11	5.01E-20	9.37E-19
