# Supplementary material for: Using Micro- and Macro-Level Network Metrics Unveils Top Communicative Gene Modules in Psoriasis
Source: Genes (Basel). 2020 Aug 10;11(8):914. doi: 10.3390/genes11080914 (PMC7464240; doi:10.3390/genes11080914)
Supplement: Supplementary file 1 [file genes-11-00914-s001.zip › supplementary_materials_version2/Figure S4.docx]

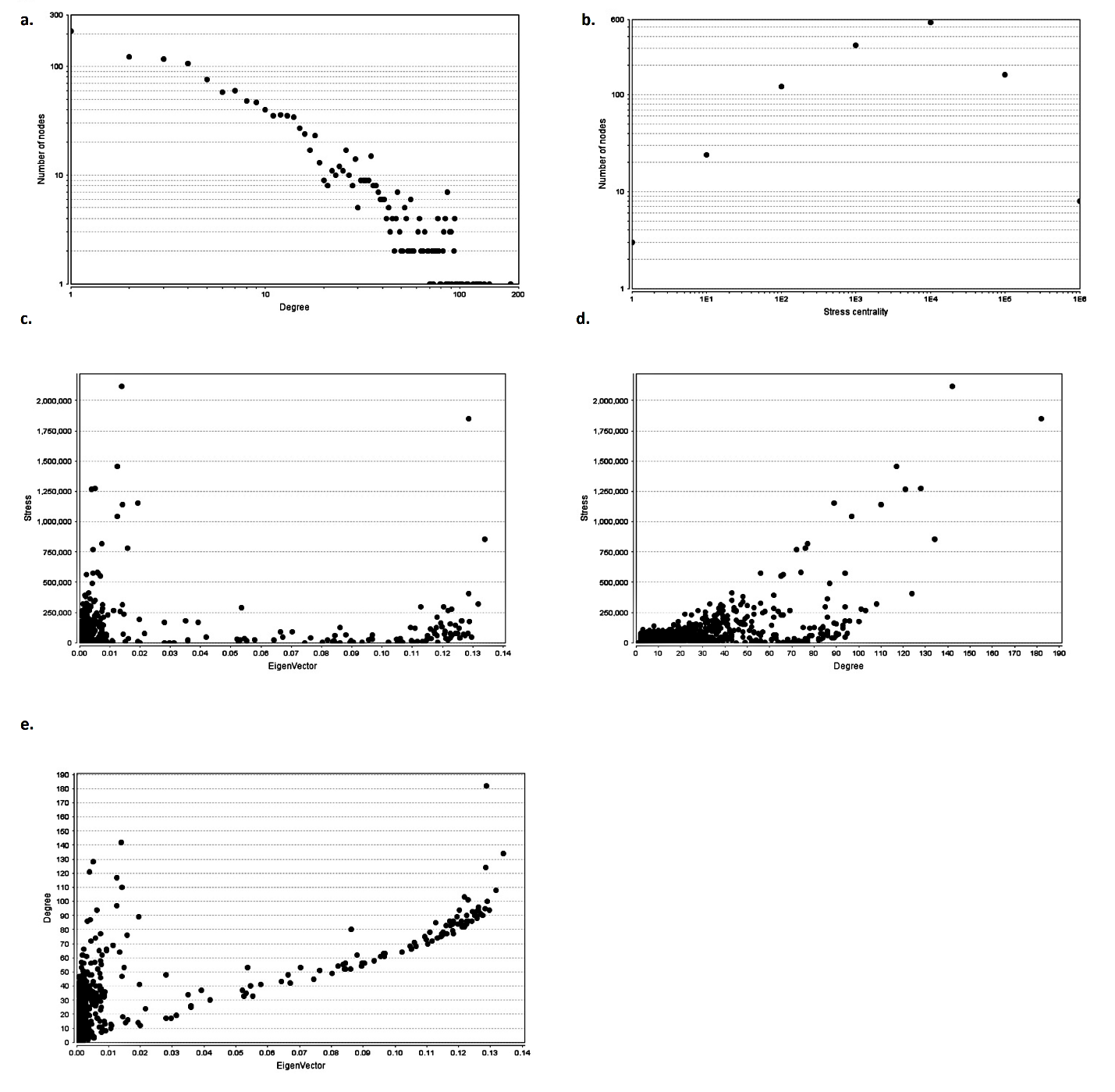


**Figure S4**. The distributions and plots of chosen metrics for 1481-gene PPI network analysis. (a) Degree distribution. (b) Stress distribution. (c) Scatter plot of stress and eigenvector. (d) Scatter plot of stress and degree. (e) Scatter plot of degree and eigenvector.
