# Supplementary material for: Using Micro- and Macro-Level Network Metrics Unveils Top Communicative Gene Modules in Psoriasis
Source: Genes (Basel). 2020 Aug 10;11(8):914. doi: 10.3390/genes11080914 (PMC7464240; doi:10.3390/genes11080914)
Supplement: Supplementary file 1 [file genes-11-00914-s001.zip › supplementary_materials_version2/Table S4.pdf]

Table S4. The characteristics of analysis on 86-gene module (module 1) based on micro level network metrics.

Metrics	Min	Max	Min (top)	Max (top)	No. of Total	Genes (descending order)
**Degree**	1	92	87	92	4	CCNB1, CDC20, CDK1, CCNA2
**Stress**	0	15636	8732	15636	6	CCNB1, MKI67, CDK1, CCNA2, FOXM1, CDC20
**Betweenness**	0	0.05	0.03	0.05	4	CCNB1, CDC20, MKI67, CDK1
**Shared genes of all groups**					5	CCNA2, CCNB1, MKI67, CDK1, CDC20
