# Supplementary material for: Using Micro- and Macro-Level Network Metrics Unveils Top Communicative Gene Modules in Psoriasis
Source: Genes (Basel). 2020 Aug 10;11(8):914. doi: 10.3390/genes11080914 (PMC7464240; doi:10.3390/genes11080914)
Supplement: Supplementary file 1 [file genes-11-00914-s001.zip › supplementary_materials_version2/Table S5.pdf]

Table S5. Main biological functions of top six communicative genes in module 1.

No.	Official Symbol	Official Full Name	Main Biological Function
**1**	CCNA2	cyclin A2	cell cycle, cell cycle G1/S phase transition, cell division, cellular response to insulin-like growth factor stimulus, cellular response to leptin stimulus, G2/M transition of mitotic cell cycle, regulation of DNA replication, histone phosphorylation, protein phosphorylation, Ras protein signal transduction
**2**	CCNB1	cyclin B1	cell cycle, regulation of cell cycle, cell division, DNA damage response, mitotic cell cycle,G2/M transition of mitotic cell cycle, mitotic metaphase plate congression, mitotic nuclear envelope disassembly, mitotic spindle organization, negative regulation of protein phosphorylation, positive regulation of attachment of spindle microtubules to kinetochore, regulation of mitotic cell cycle spindle assembly checkpoint, T cell homeostasis, tissue regeneration
**3**	MKI67	marker of proliferation Ki-67	cell proliferation
**4**	CDK1	cyclin dependent kinase 1	cell cycle, regulation of cell cycle, cell division, cell proliferation, cell aging, apoptotic process, mitotic cell cycle, mitotic cell cycle phase transition, mitotic G2 DNA damage checkpoint, mitotic G2/M transition checkpoint, activation of MAPK activity, anaphase-promoting complex, DNA damage response, DNA repair, DNA replication,G1/S

			transition of mitotic cell cycle,G2/M transition of mitotic cell cycle
**5**	CDC20	cell division cycle 20	cell cycle, cell differentiation, cell division, anaphase promoting complex-dependent catabolic process, mitotic spindle assembly, mitotic spindle assembly checkpoint, positive regulation of cell proliferation, protein ubiquitination, regulation of mitotic cell cycle phase transition
**6**	FOXM1	forkhead box M1	cell cycle, cellular response to DNA damage stimulus, DNA repair, G2/M transition of mitotic cell cycle, negative regulation of cell aging, regulation of cell cycle, regulation of cell cycle arrest, regulation of cell proliferation, positive regulation of transcription by RNA polymerase II, negative regulation of transcription by RNA polymerase II
