# Supplementary material for: Using Micro- and Macro-Level Network Metrics Unveils Top Communicative Gene Modules in Psoriasis
Source: Genes (Basel). 2020 Aug 10;11(8):914. doi: 10.3390/genes11080914 (PMC7464240; doi:10.3390/genes11080914)
Supplement: Supplementary file 1 [file genes-11-00914-s001.zip › supplementary_materials_version2/Table S7.pdf]

Table S7. Top 10 enriched pathways in module 1 in order of combined score by WikiPathways 2019 HUMAN database

Index	Term	Genes	Combined Score
**1**	Regulation of sister chromatid separation at the metaphase-anaphase transition WP4240	CDC20;CENPE;PTTG1;BUB1B;BUB1; MAD2L1	2268.4
**2**	Retinoblastoma Gene in Cancer WP2446	TOP2A;CCNA2;CDT1;CCNB2;CCNB1; RRM2;ORC1;KIF4A;CHEK1;CDK1;TTK; CDC25A	1079.0
**3**	Cell Cycle WP179	PLK1;TTK;CDC6;CDC25C;CDC25A;CDC20; CCNA2;CCNB2;CCNB1;ORC1;PTTG1; CHEK1; CDK1;BUB1	996.1
**4**	Gastric Cancer Network 1 WP2361	TOP2A;TPX2;CENPF;ECT2;AURKA;KIF15	956.5
**5**	ATM Signaling Pathway WP2516	CCNB1;CHEK1;CDK1;CDC25C;CDC25A	409.4
**6**	Integrated Cancer Pathway WP1971	BLM;CHEK1;PLK1;CDK1;CDC25A	359.3
**7**	DNA Damage Response WP707	CCNB2;CCNB1;CHEK1;CDK1;CDC25C; CDC25A	299.0
**8**	miRNA Regulation of DNA Damage Response WP1530	CCNB2;CCNB1;CHEK1;CDK1;CDC25C; CDC25A	281.3
**9**	DNA IR-damage and cellular response via ATR WP4016	EXO1;CHEK1;PLK1;CDK1;FOXM1; CDC25C	237.3
**10**	DNA Replication WP466	CDT1;ORC1;MCM10;CDC6	229.5
