# Supplementary material for: Using Micro- and Macro-Level Network Metrics Unveils Top Communicative Gene Modules in Psoriasis
Source: Genes (Basel). 2020 Aug 10;11(8):914. doi: 10.3390/genes11080914 (PMC7464240; doi:10.3390/genes11080914)
Supplement: Supplementary file 1 [file genes-11-00914-s001.zip › supplementary_materials_version2/Table S6.pdf]

Table S6. Top 10 enriched pathways in module 1 ranked in order of combined score by KEGG 2019 HUMAN Database

index	Term	Genes	Combined score
**1**	Cell Cycle	PLK1;BUB1B;TTK;CDC6;CDC25C;CDC25A; CDC20;CCNA2;CCNB2;CCNB1;ORC1;PTTG1; CHEK1; CDK1;BUB1;MAD2L1	1307.85
**2**	Progesterone-mediated oocyte maturation	CCNA2;CCNB2;CCNB1;PLK1;CDK1;CDC25C; BUB1;CDC25A;MAD2L1;AURKA	586.43
**3**	Oocyte meiosis	CDC20;CCNB2;CCNB1;PTTG1;PLK1;CDK1; CDC25C;BUB1;MAD2L1;AURKA	420.88
**4**	p53 signaling pathway	CCNB2;CCNB1;RRM2;CHEK1;CDK1;GTSE1	275.82
**5**	Cellular senescence	CCNA2;CCNB2;CCNB1;CHEK1;CDK1;FOXM1; CDC25A	122.65
**6**	Human T-cell leukemia virus 1 infection	CDC20;CCNB2;CCNA2; BUB1;PTTG1; CHEK1; BUB1B; MAD2L1	74.52
**7**	Human immunodeficiency virus 1 infection	CCNB2;CCNB1; CHEK1; CDC25C;CDK1	33.52
	Pyrimidine metabolism	RRM2;TK1	30.09
**8**	Mismatch repair	EXO1	23.86
**9**	FoxO signaling pathway	CCNB2;CCNB1;PLK1	20.85
