# Supplementary material for: Using Micro- and Macro-Level Network Metrics Unveils Top Communicative Gene Modules in Psoriasis
Source: Genes (Basel). 2020 Aug 10;11(8):914. doi: 10.3390/genes11080914 (PMC7464240; doi:10.3390/genes11080914)
Supplement: Supplementary file 1 [file genes-11-00914-s001.zip › supplementary_materials_version2/Table S8.pdf]

Table S8. The characteristics of analysis on 63-gene module (module 2) based on micro level network metrics.

Metrics	Min	Max	Ave	Min (top)	Max (top)	Total	Genes (descending order)
**Degree**	1	43	15.07	26	43	9	H2AFX, STAT3, EGF, IL1B, STAT1, IFNG, CXCL8, MAPK14, CXCL10
**Stress**	0	29876	2.67e+03	8518	29876	4	STAT3, EGF, MAPK14, IL1B
**Eigenvector**	0.000003	0.05	0.003	0.007	0.05	6	H2AFX, MCL1, STAT3, EGF, UBE2N, MAPK14
**Betweenness**	0	0.07	0.007	0.04	0.07	3	EGF, STAT3, MAPK14
**Shared genes of all groups**						2	EGF, STAT3
