# Supplementary material for: Using Micro- and Macro-Level Network Metrics Unveils Top Communicative Gene Modules in Psoriasis
Source: Genes (Basel). 2020 Aug 10;11(8):914. doi: 10.3390/genes11080914 (PMC7464240; doi:10.3390/genes11080914)
Supplement: Supplementary file 1 [file genes-11-00914-s001.zip › supplementary_materials_version2/Table S9.pdf]

Table S9. Main biological functions of 11 top communicative genes in module 2.

index	Official Symbol	Official Full Name	Main Function
**1**	STAT3	signal transducer and activator of transcription 3	acute-phase response, regulation of cell cycle, cell proliferation, cellular response to cytokine stimulus, cytokine-mediated signaling pathway, inflammatory response,interleukin-23-mediated signaling pathway, JAK-STAT cascade, negative regulation of cell death, positive regulation of NF-kappaB transcription factor activity, T-helper 17 cell lineage, commitment, cellular response to leptin stimulus,interleukin-6-mediated signaling pathway, mRNA transcription by RNA polymerase II, regulation of transcription by RNA polymerase II, viral process
**2**	H2AFX	H2A histone family member X	cell cycle, cellular response to DNA damage stimulus, DNA repair, viral process
**3**	EGF	epidermal growth factor	activation of MAPK activity, epidermal growth factor receptor signaling pathway, ERK1 and ERK2 cascade, regulation of JAK-STAT cascade, regulation of signaling receptor activity, signal transduction, regulation of protein transport, regulation of protein secretion, positive regulation of ubiquitin-dependent protein catabolic process
**4**	IL1B	interleukin 1 beta	activation of MAPK activity, acute inflammatory response, apoptotic process, cytokine-mediated signaling pathway, immune response ,inflammatory response,interleukin-1 beta

			production,interleukin-1-mediated signaling pathway, interleukin-6 production, neutrophil activation, neutrophil chemotaxis, leukocyte migration, macrophage chemotaxis, monocyte aggregation, negative regulation of cell proliferation, negative regulation of gene expression, negative regulation of transcription by RNA polymerase II, positive regulation of cell division, positive regulation of immature T cell proliferation in thymus, positive regulation of interleukin-8 production, positive regulation of NF-kappaB transcription factor activity, positive regulation of T cell mediated immunity, positive regulation of T cell proliferation, positive regulation of T-helper 1 cell cytokine production, response to interleukin-1,response to virus.
**5**	STAT1	signal transducer and activator of transcription 1	cellular response to cytokine stimulus, cellular response to interferon-beta,cytokine-mediated signaling pathway, response to cytokine, JAK-STAT cascade, negative regulation of transcription by RNA polymerase II, positive regulation of interferon-alpha, defense response to virus
**6**	IFNG	interferon gamma	apoptotic process , cell cycle arrest, cell surface receptor signaling pathway, defense response to virus, extrinsic apoptotic signaling pathway, interferon-gamma-mediated signaling pathway , interleukin-12-mediated signaling pathway, JAK-STAT cascade , microglial cell activation, negative regulation of gene

			expression, negative regulation of interleukin-17 production, negative regulation of tau-protein kinase activity, negative regulation of transcription by RNA polymerase II, negative regulation of transcription, positive regulation of amyloid-beta formation, positive regulation of autophagy, positive regulation of cell proliferation
**7**	CXCL8	C-X-C motif chemokine ligand 8	cell cycle arrest, cellular response to fibroblast growth factor stimulus, cellular response to interleukin-1,cellular response to tumor necrosis factor, cytokine-mediated signaling pathway, inflammatory response, positive regulation of neutrophil chemotaxis, neutrophil activation, positive regulation of angiogenesis
**8**	MAPK14	mitogen-activated protein kinase 14	3'-UTR-mediated mRNA stabilization, activation of MAPK activity, apoptotic process,c ell morphogenesis, cell motility, cellular response to tumor necrosis factor, cellular response to virus, chemotaxis, neutrophil degranulation, p38MAPK cascade, positive regulation of macrophage chemotaxis, positive regulation of transcription by RNA polymerase II, regulation of cytokine production involved in inflammatory response, Ras protein signal transduction, transmembrane receptor protein serine/threonine kinase signaling pathway
**9**	MCL1	MCL1 apoptosis regulator, BCL2 family member	apoptotic process, cell differentiation, cell fate determination, cytokine-mediated

			signaling pathway, negative regulation of autophagy, response to cytokine
**10**	UBE2N	ubiquitin conjugating enzyme E2 N	activation of MAPK activity, cellular protein modification process, cellular response to DNA damage stimulus, DNA repair,interleukin-1-mediated signaling pathway, JNK cascade, positive regulation of I-kappaB kinase/NF-kappaB signaling,T cell receptor signaling pathway, ubiquitin-dependent protein catabolic process
**11**	CXCL10		adenylate cyclase-activating G protein-coupled receptor signaling pathway, blood circulation, cell-cell signaling, cell surface receptor signaling pathway, CXCR3 chemokine receptor binding, chemokine activity, chemoattractant activity, cAMP-dependent protein kinase regulator activity, T cell chemotaxis, regulation of T cell chemotaxis, regulation of cell proliferation, regulation of apoptotic process, positive regulation of T cell migration, cytokine-mediated signaling pathway, chemokine-mediated signaling pathway
