# Supplementary material for: Using Micro- and Macro-Level Network Metrics Unveils Top Communicative Gene Modules in Psoriasis
Source: Genes (Basel). 2020 Aug 10;11(8):914. doi: 10.3390/genes11080914 (PMC7464240; doi:10.3390/genes11080914)
Supplement: Supplementary file 1 [file genes-11-00914-s001.zip › supplementary_materials_version2/Figure S6.docx]

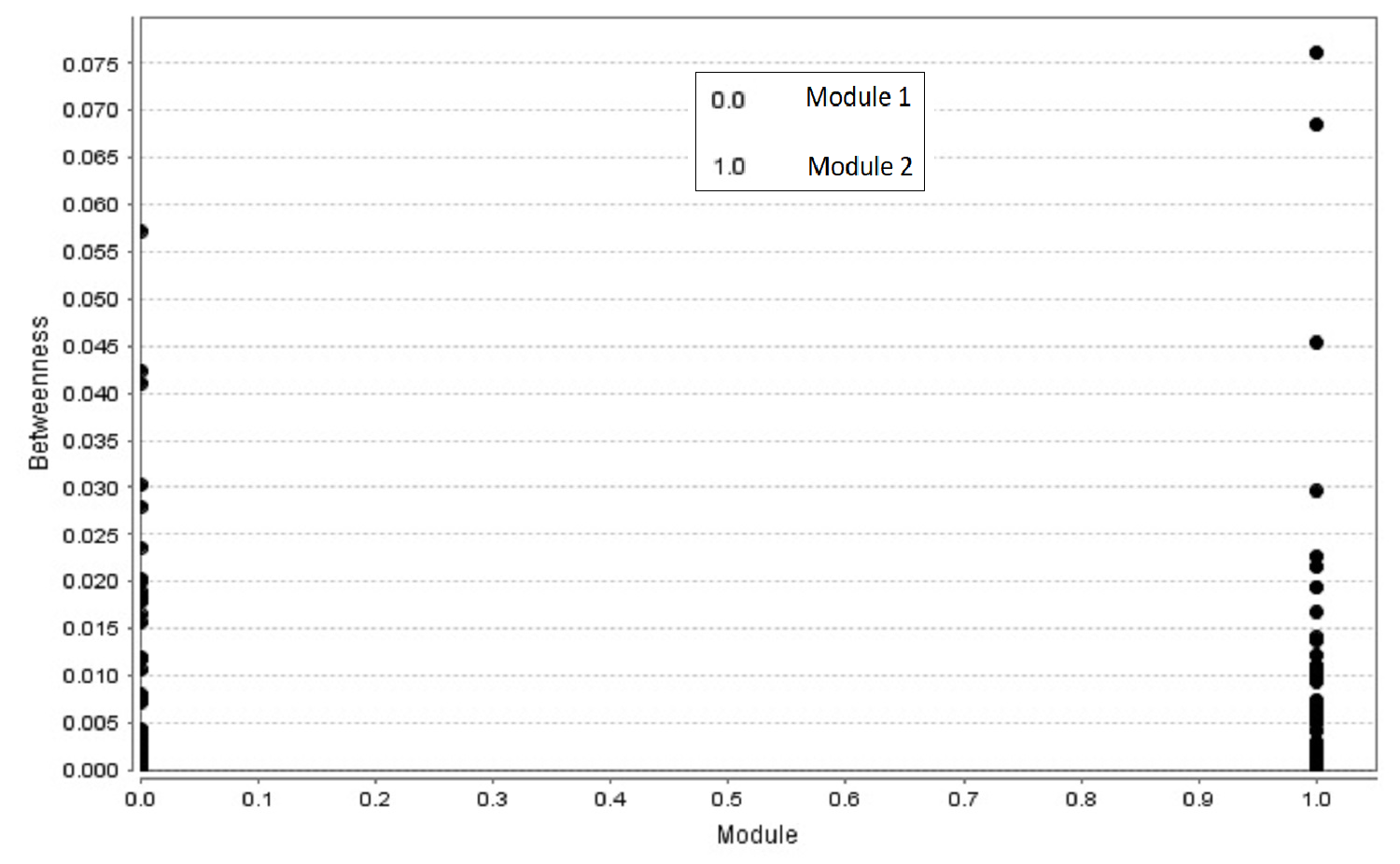


**Figure S6**. Metrics distribution for each module. (a) Betweenness distribution.


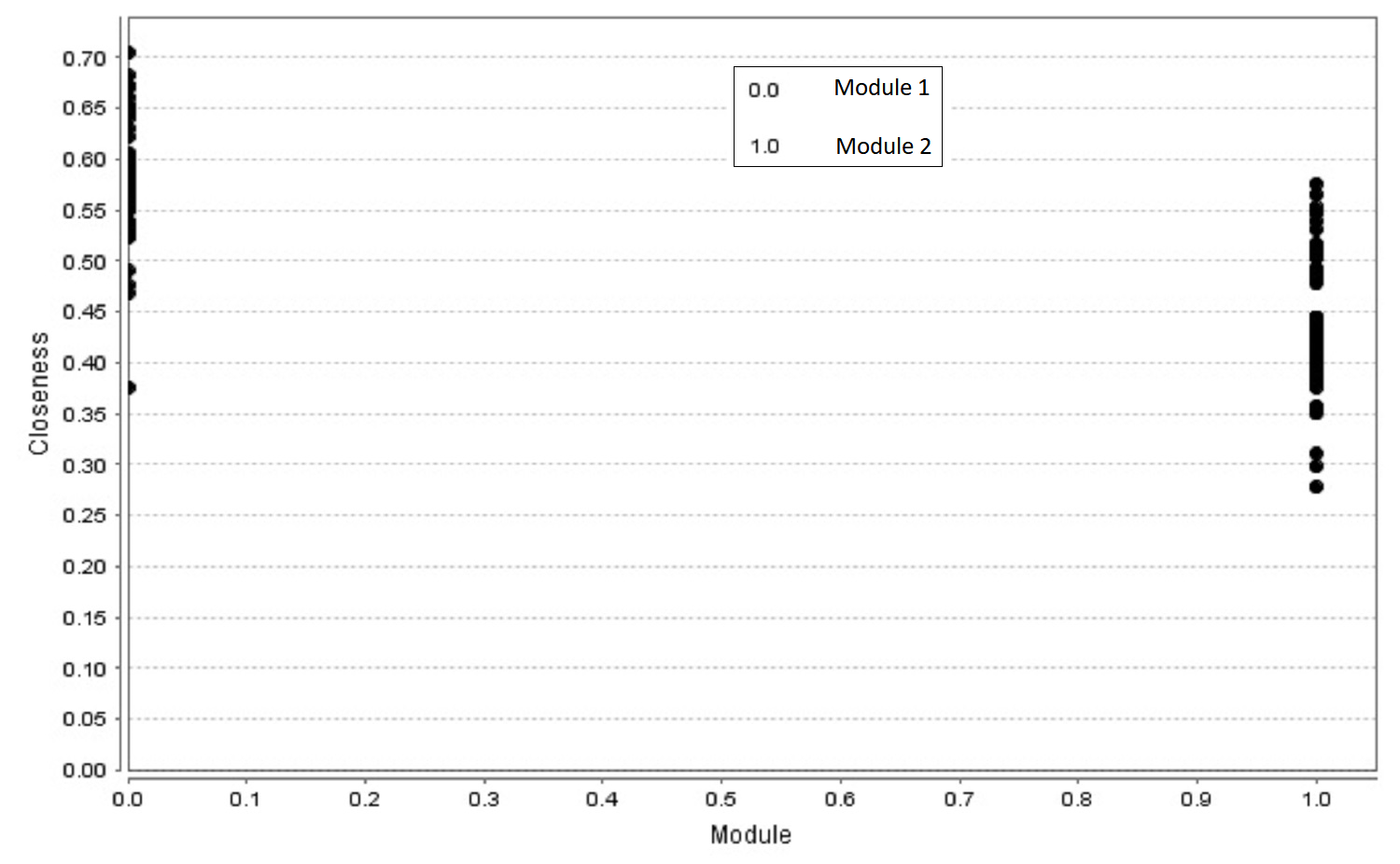


**Figure S6.** Metrics distribution for each module. (b) Closeness distribution.


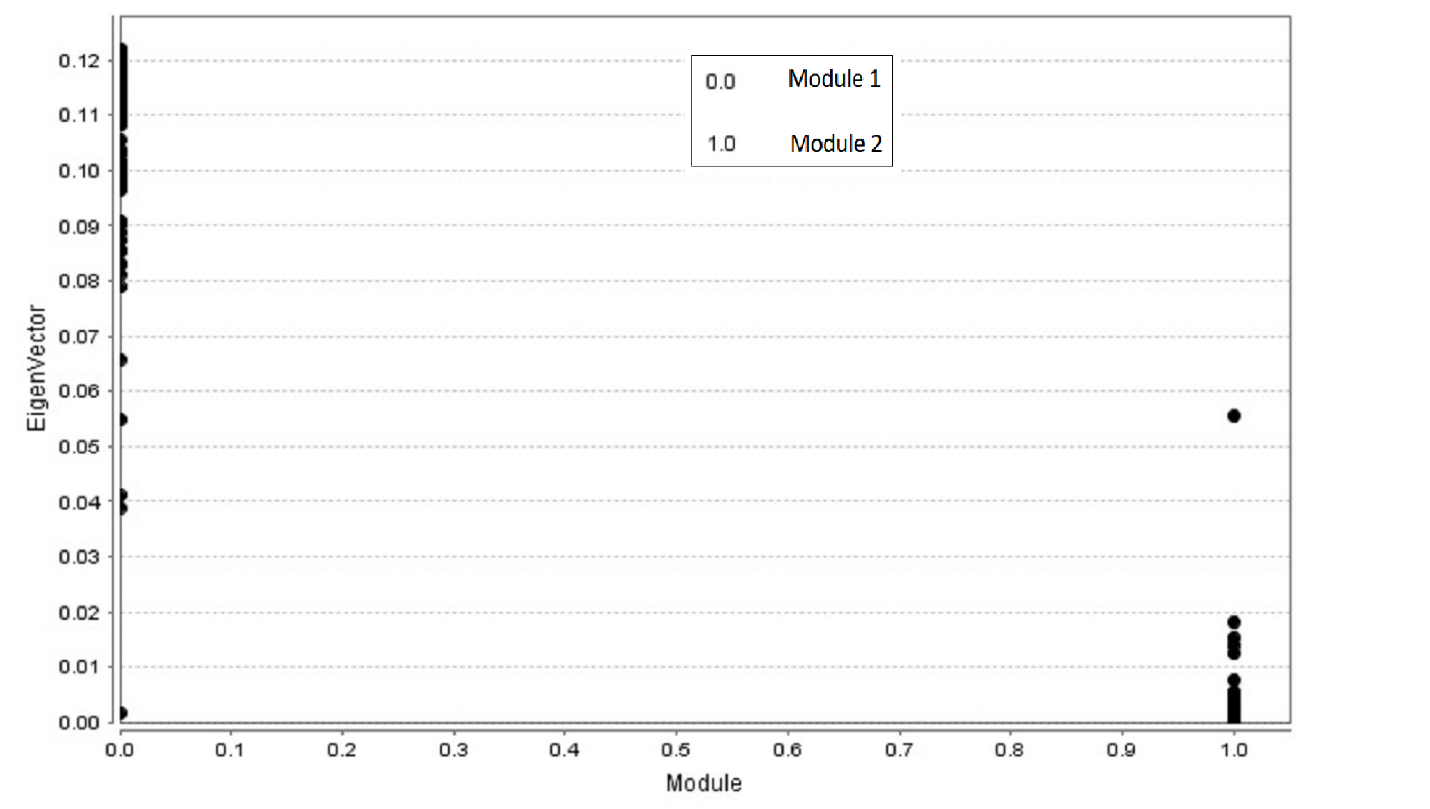


**Figure S6**. Metrics distribution for each module. (c) Eigenvector distribution.


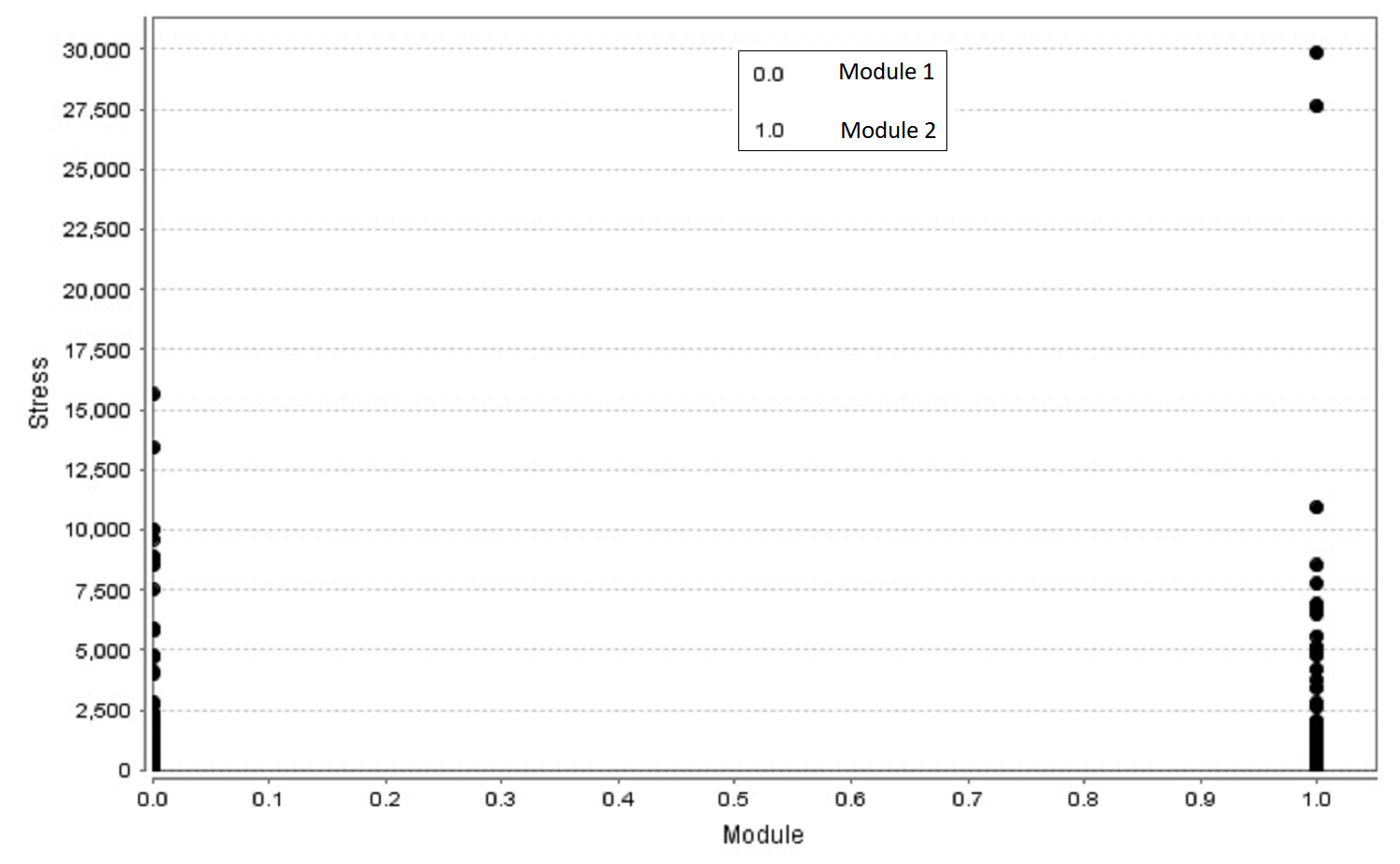


**Figure S6**. Metrics distribution for each module. (d) Stress distribution.


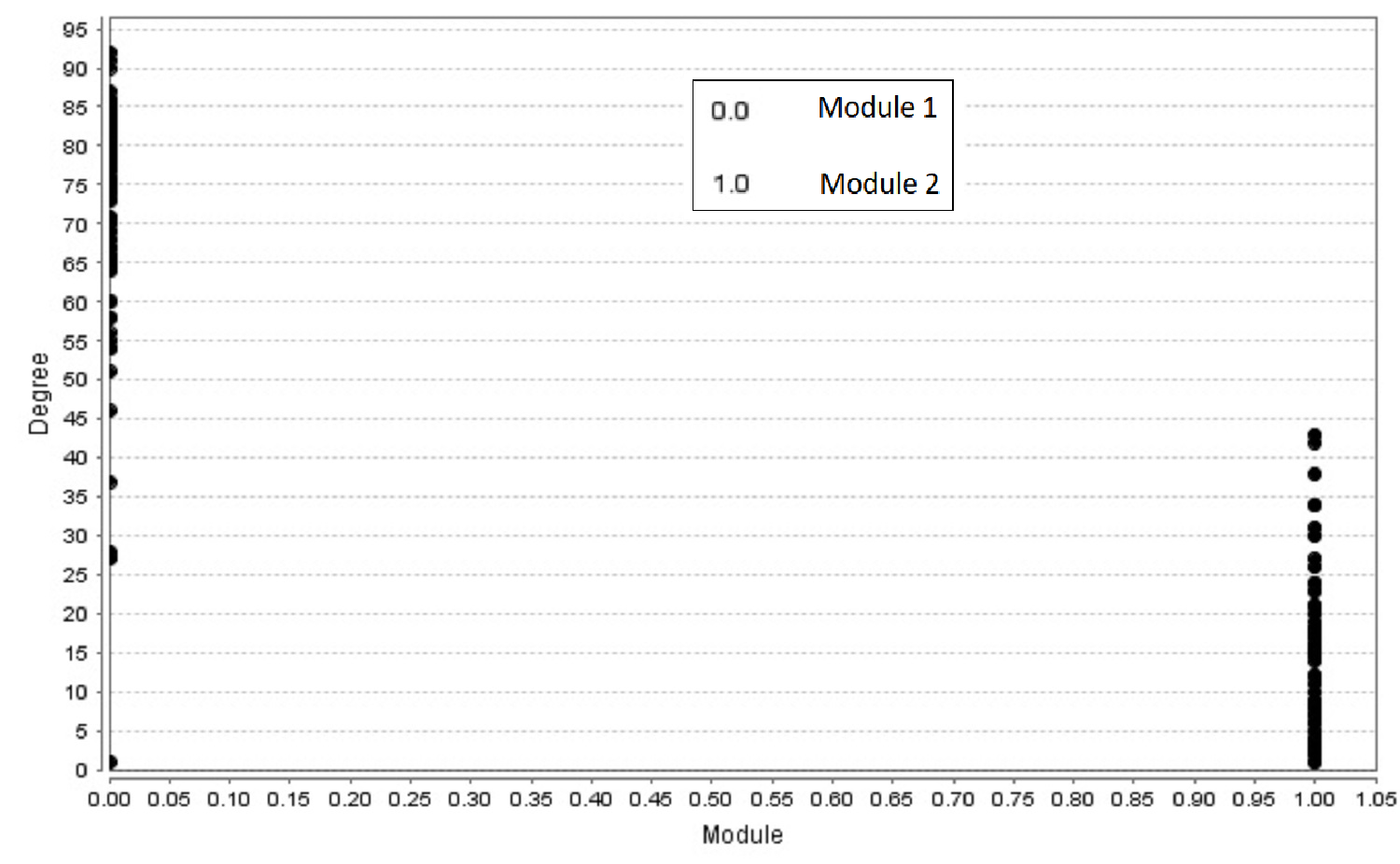


**Figure S6**. Metrics distribution for each module. (e) Degree distribution.
