# Supplementary material for: Using Micro- and Macro-Level Network Metrics Unveils Top Communicative Gene Modules in Psoriasis
Source: Genes (Basel). 2020 Aug 10;11(8):914. doi: 10.3390/genes11080914 (PMC7464240; doi:10.3390/genes11080914)
Supplement: Supplementary file 1 [file genes-11-00914-s001.zip › supplementary_materials_version2/Table S14.pdf]

**Table S14.** 149 primary communicative genes

| Module 1 (cell cycle) | | Module 2 (immune system) | |
|---|---|---|---|
| **GeneSymbole** | **LogFC** | **GeneSymbole** | **LogFC** |
| CCNB1 | 1.751606 | EGF | 2.015857 |
| CDC20 | 1.018947 | H2AFX | 1.339597 |
| CDK1 | 1.782422 | IFNG | 3.903068 |
| CCNA2 | 1.425815 | MAPK14 | 1.380447 |
| AURKA | 1.469726 | SOCS3 | 1.222717 |
| TPX2 | 1.218712 | IRF1 | 1.246018 |
| BIRC5 | 1.666018 | CAT | -1.02214 |
| ASPM | 1.459225 | IL1B | 1.24602 |
| BUB1B | 1.14086 | STAT1 | 2.268373 |
| PLK1 | 1.081502 | DDX58 | 1.732727 |
| MKI67 | 1.555337 | MCL1 | 1.374379 |
| KIF4A | 1.435557 | NCAM1 | -1.50577 |
| BUB1 | 1.347923 | CXCL8 | 1.938556 |
| CENPF | 1.312028 | CDH2 | -1.19601 |
| CCNB2 | 1.265481 | UBE2N | 1.454899 |
| KIF2C | 1.063634 | LYN | 1.202117 |
| CENPE | 1.727115 | IFIH1 | 2.025857 |
| TTK | 1.547076 | CXCL10 | 2.492372 |
| EXO1 | 1.452831 | OASL | 3.850159 |
| HMMR | 1.439402 | UBE2L6 | 1.137636 |
| DLGAP5 | 1.314865 | IL17A | 2.007943 |
| TOP2A | 1.270339 | MUC1 | -1.87438 |
| KIF11 | 1.072411 | NEDD4L | -1.13571 |
| MAD2L1 | 1.171882 | PIK3R1 | -1.11169 |
| KIF15 | 1.106039 | ISG15 | 2.421688 |
| NUF2 | 1.048822 | ACTA2 | -1.07901 |
| RRM2 | 2.435553 | HERC6 | 1.267729 |
| NDC80 | 1.600941 | FGFR1 | 1.039025 |
| KIF20A | 1.539143 | PRKCB | -1.20835 |
| NCAPG | 1.411422 | IRF7 | 1.608537 |
| KIF23 | 1.296159 | NOS2 | 2.907845 |
| CDCA5 | 1.031743 | PPARG | -1.33643 |
| MELK | 1.531124 | LEP | -1.82199 |
| CEP55 | 1.342255 | CBL | 1.327704 |
| NCAPH | 1.23415 | CXCL9 | 1.938556 |
| NEK2 | 1.205286 | HMOX1 | -2.42017 |
| PBK | 1.200299 | APOE | -1.11821 |
| PRC1 | 1.112868 | CXCL11 | 1.527269 |
| CDC6 | 1.500884 | PDGFRA | -1.04914 |
| CDC25C | 1.138681 | OAS1 | 2.522087 |
| OIP5 | 1.048265 | OAS2 | 2.865296 |
| CDCA2 | 1.422932 | OAS3 | 2.350212 |
| **DTL** | 1.127632 | IL23A | 1.24602 |

| | | | |
|---|---|---|---|
| FOXM1 | -1.00236 | PPARA | -1.00535 |
| CHEK1 | 1.602945 | REN | 3.407901 |
| MCM10 | 1.532975 | RALA | 1.081825 |
| TRIP13 | 1.273851 | CALML5 | 1.053977 |
| CDKN3 | 1.425817 | CALML3 | 1.267862 |
| CENPN | 1.357144 | CFTR | -1.2463 |
| PTTG1 | 1.235721 | MAPK13 | 1.107849 |
| RAD51AP1 | 1.002493 | DLG2 | 1.029973 |
| KIF18A | 1.290972 | DLG3 | 1.668509 |
| ASF1B | 1.259889 | DCN | 1.216145 |
| SKA3 | 1.113315 | TJP1 | 1.551018 |
| DEPDC1 | 1.834209 | SPTAN1 | -1.36387 |
| KIF14 | 1.52683 | CYP2B6 | -1.15122 |
| ECT2 | -1.62846 | MYH14 | -1.43884 |
| SMC4 | 1.037946 | GNAZ | 1.054781 |
| SPC25 | 1.542773 | ACACB | -1.32206 |
| SKA1 | 1.395567 | HSD11B1 | 1.526098 |
| KIF18B | 1.257541 | RIT1 | 1.168371 |
| CDT1 | 1.287663 | MGP | -1.0137 |
| CKS2 | 1.166541 | STAT3 | 1.519357 |
| GTSE1 | 1.20401 | | |
| SGOL1 | 1.119508 | | |
| ERCC6L | 1.371814 | | |
| TROAP | 1.278355 | | |
| ORC1 | 1.074018 | | |
| CDC25A | 1.04232 | | |
| CASC5 | 1.269604 | | |
| CKAP2L | 1.097901 | | |
| FAM83D | -1.21593 | | |
| KPNA2 | 1.160963 | | |
| CENPI | 1.003546 | | |
| HELLS | 2.781395 | | |
| CCNF | 1.333266 | | |
| TK1 | 1.035878 | | |
| DEPDC1B | -1.1314 | | |
| STIL | 1.197017 | | |
| PARPBP | 1.096107 | | |
| TICRR | 1.1655 | | |
| BLM | 1.13553 | | |
| KIF2A | 1.066713 | | |
| NDEL1 | 1.06672 | | |
| TUBG1 | 1.230465 | | |
| TNNC1 | -1.64639 | | |
