# Supplementary material for: Using Micro- and Macro-Level Network Metrics Unveils Top Communicative Gene Modules in Psoriasis
Source: Genes (Basel). 2020 Aug 10;11(8):914. doi: 10.3390/genes11080914 (PMC7464240; doi:10.3390/genes11080914)
Supplement: Supplementary file 1 [file genes-11-00914-s001.zip › supplementary_materials_version2/Figure S1.pdf]

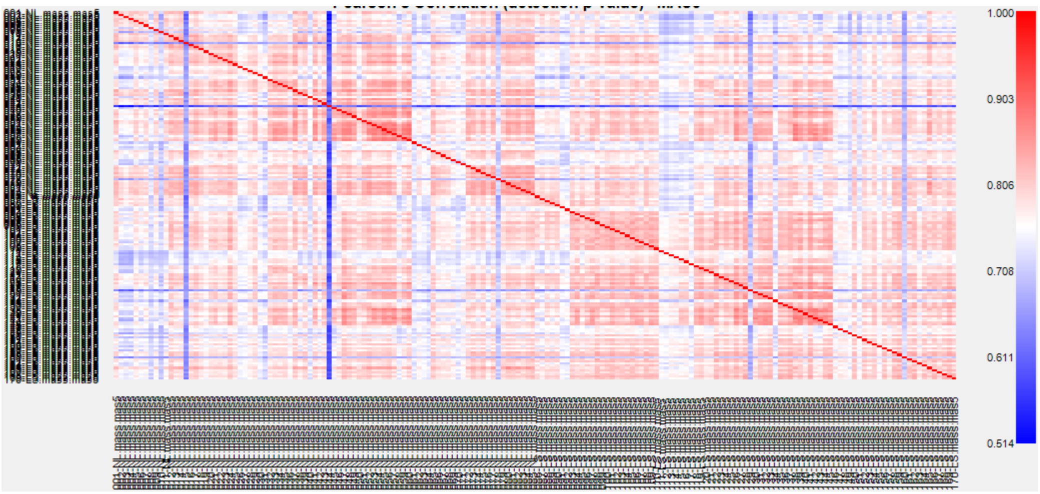

Figure S1. The heatmap of the Pearson's correlation coefficient (r2) of 170 samples as pairwise comparisons for quality control. The r2 values have been converted into pseudo-color scale ranging from 0.5 to 1. Pearson correlation coefficient is a measure of the degree of linear relationship between two samples. A higher correlation indicates a more similar gene expression pattern between two samples. Each row or column represents one sample. Each point is a Pearson correlation coefficient between a sample in a column and a sample in a row. Darker blue and red indicate lower and higher values of r2, respectively. All 170 data points in one row (same for one column) show the correlation of one sample to all other samples. Thus, a blue line conveys that the sample does not correlate well to other samples. We considered a threshold of 0.514 as the minimum accepted value (Rule of Thumb*) for a significant linear relationship between samples.

* T. C. Krehbiel, "Correlation Coefficient Rule of Thumb," *Decis. Sci. J. Innov. Educ.*, vol. 2, no. 1, pp. 97–100, 2004.
