# Supplementary material for: Using Micro- and Macro-Level Network Metrics Unveils Top Communicative Gene Modules in Psoriasis
Source: Genes (Basel). 2020 Aug 10;11(8):914. doi: 10.3390/genes11080914 (PMC7464240; doi:10.3390/genes11080914)
Supplement: Supplementary file 1 [file genes-11-00914-s001.zip › supplementary_materials_version2/Figure S2.pdf]

a

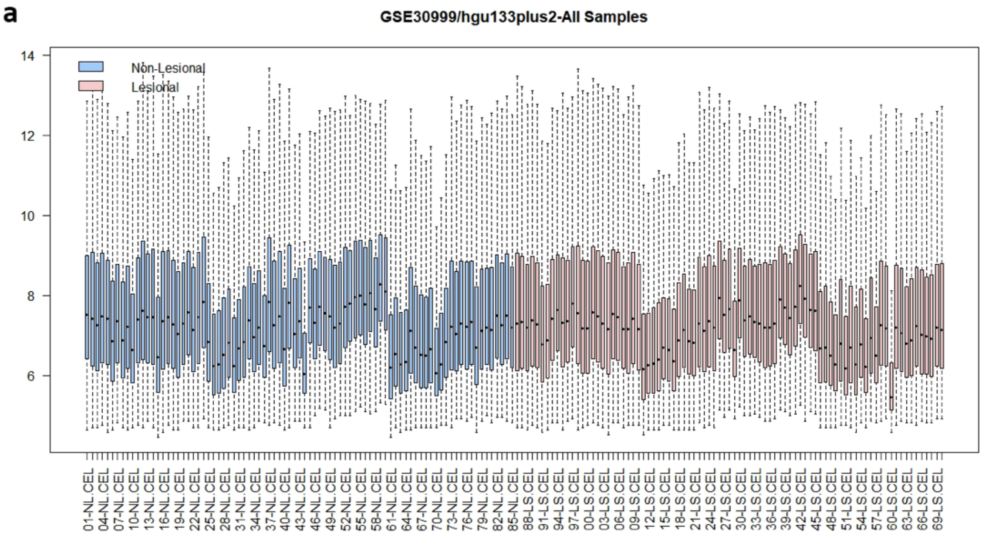

b

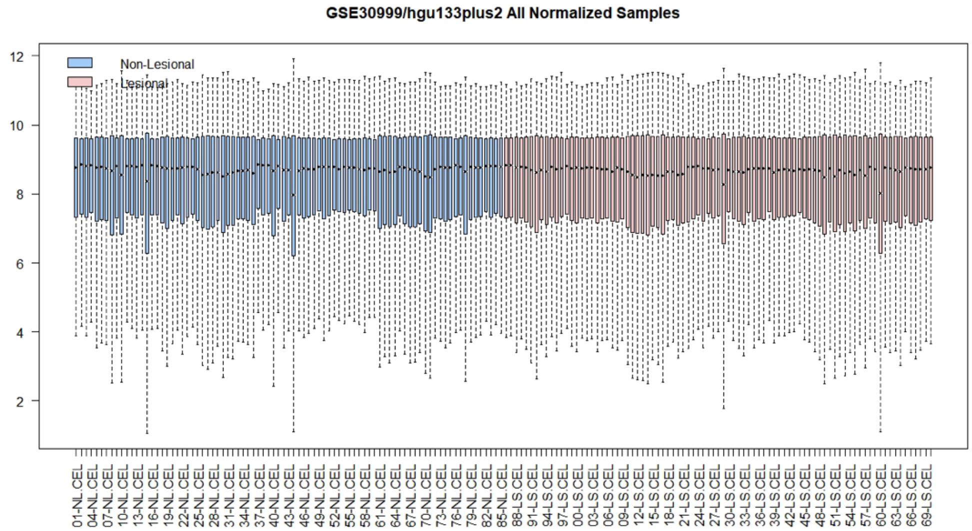

Figure S2. Boxplots of raw data for the expression level values transformed to log base 2. (a) And (b) demonstrate pre-normalized and normalized samples, respectively.
