# Supplementary material for: Using Micro- and Macro-Level Network Metrics Unveils Top Communicative Gene Modules in Psoriasis
Source: Genes (Basel). 2020 Aug 10;11(8):914. doi: 10.3390/genes11080914 (PMC7464240; doi:10.3390/genes11080914)
Supplement: Supplementary file 1 [file genes-11-00914-s001.zip › supplementary_materials_version2/Figure S3.pdf]

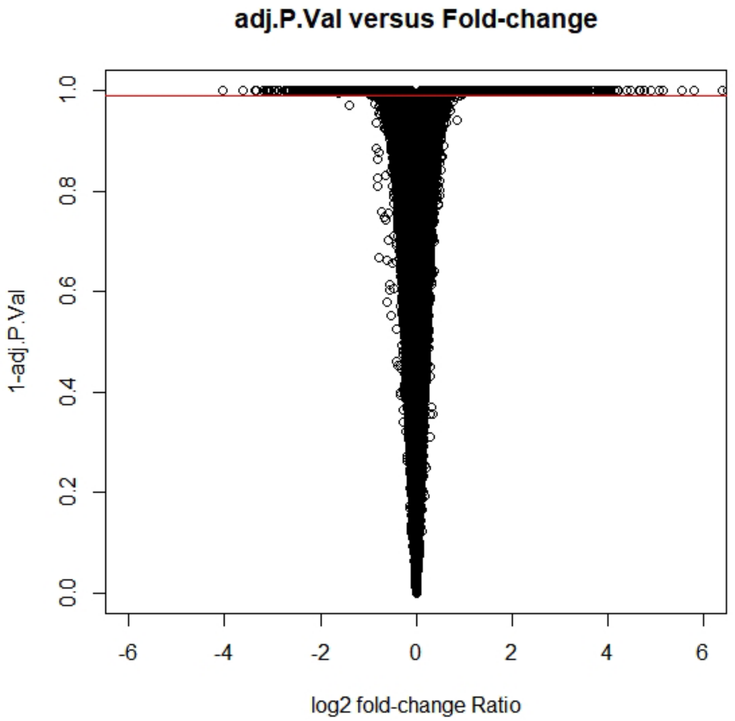

Figure S3. Volcano plot representing the result of hypothesis testing between lesional and non-lesional samples. The vertical axis corresponds to 1 - adjusted p-value, and the horizontal axis displays the log base 2 fold change values between two groups. The red line shows where (1 - adjusted p-value) = 0.01 with points above the red line having adjusted p-value < 0.01. Each point on the plot indicates the statistical result for a single gene.
