# Supplementary material for: Using Micro- and Macro-Level Network Metrics Unveils Top Communicative Gene Modules in Psoriasis
Source: Genes (Basel). 2020 Aug 10;11(8):914. doi: 10.3390/genes11080914 (PMC7464240; doi:10.3390/genes11080914)
Supplement: Supplementary file 1 [file genes-11-00914-s001.zip › supplementary_materials_version2/Table S12.pdf]

Table S12. Intersectional pathways among top 20 enriched pathways based on 149 merged genes from modules 1 and 2 sorted by combined score by KEGG 2019 HUMAN Database

Index	Term	Genes	Combined score
**1**	Progesterone-mediated oocyte maturation	PLK1;PIK3R1;CDC25C;MAPK14;CDC25A; AURKA;MAPK13;CCNA2;CCNB2;CCNB1; CDK1;BUB1;MAD2L1	500.8
**2**	Influenza A	CXCL8;PRKCB;DDX58;STAT1;PIK3R1; MAPK14;MAPK13;IFIH1;SOCS3;CXCL10; IFNG; OAS1;OAS2;IL1B;OAS3;IRF7;KPNA2	428.6
**3**	Oocyte meiosis	CDC20;CCNB2;CCNB1;CALML5;PTTG1;PLK1 ;CDK1;CALML3;CDC25C;BUB1;AURKA; MAD2L1	290.6
**4**	Cellular senescence	CXCL8;CALML5;CALML3;PIK3R1;FOXM1; MAPK14;CDC25A;MAPK13;CCNA2;CCNB2; CCNB1;CHEK1;CDK1	242.5
**5**	Epstein-Barr virus infection	LYN;DDX58;STAT1;STAT3;ISG15;PIK3R1; MAPK14;MAPK13;CCNA2;CXCL10;OAS1; OAS2;OAS3;IRF7	203.9
**6**	Hepatitis B	IFIH1;CCNA2;CXCL8;PRKCB;DDX58;STAT1; STAT3;IRF7;BIRC5;PIK3R1;MAPK14; MAPK13	192.5
**7**	FoxO signaling pathway	CCNB2;CCNB1;EGF;PLK1;CAT;STAT3; PIK3R1;MAPK14;MAPK13	130.4
