# Supplementary material for: Using Micro- and Macro-Level Network Metrics Unveils Top Communicative Gene Modules in Psoriasis
Source: Genes (Basel). 2020 Aug 10;11(8):914. doi: 10.3390/genes11080914 (PMC7464240; doi:10.3390/genes11080914)
Supplement: Supplementary file 1 [file genes-11-00914-s001.zip › supplementary_materials_version2/Figure S6.pdf]

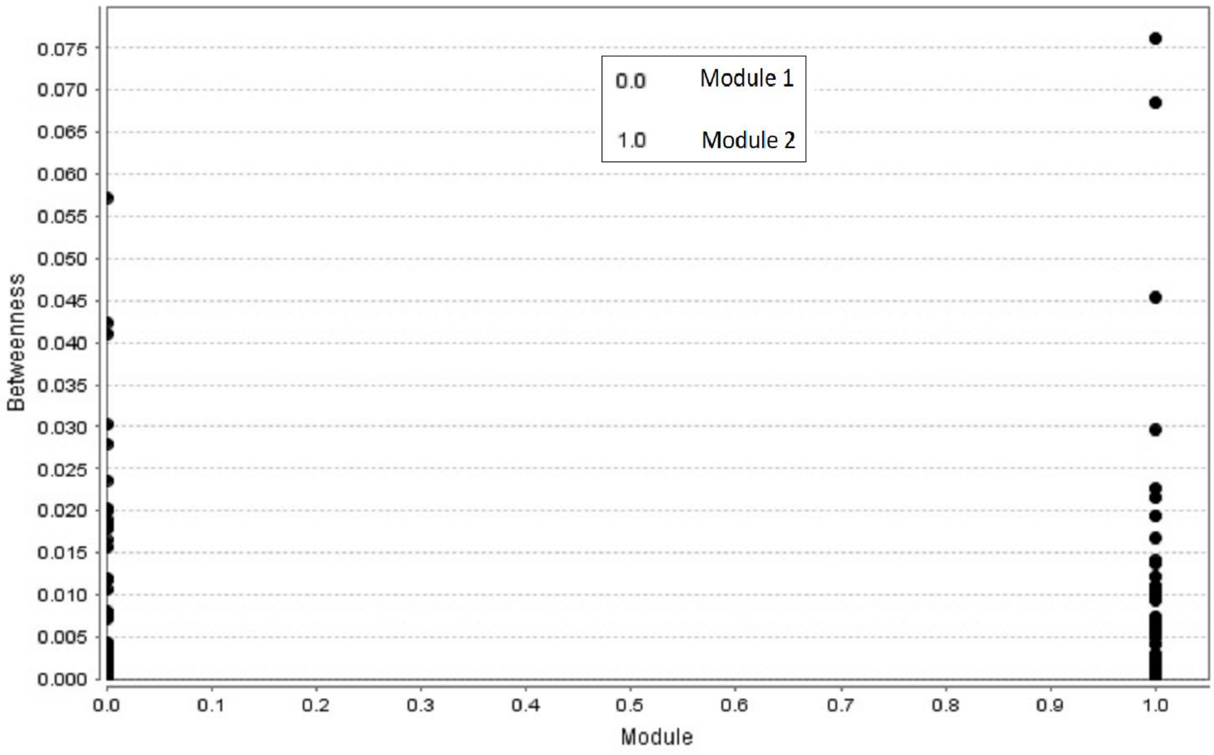

Figure S6. Metrics distribution for each module. (a) Betweenness distribution.

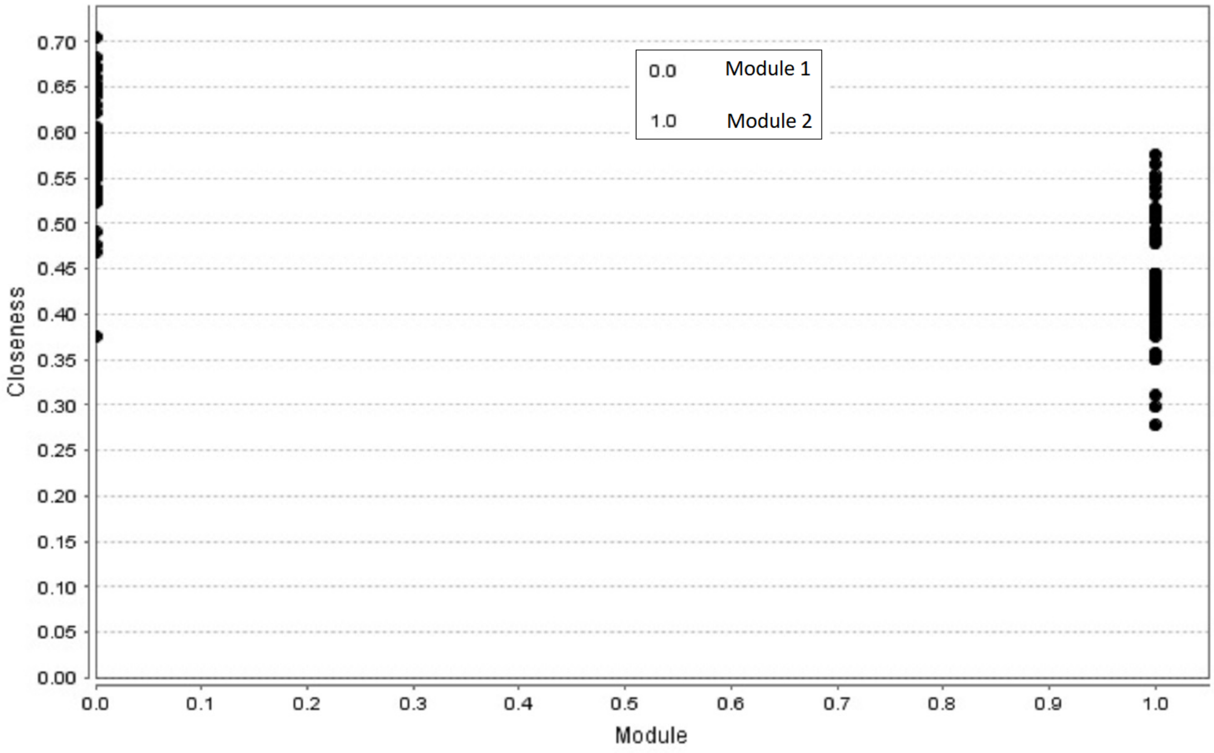

Figure S6. Metrics distribution for each module. (b) Closeness distribution.

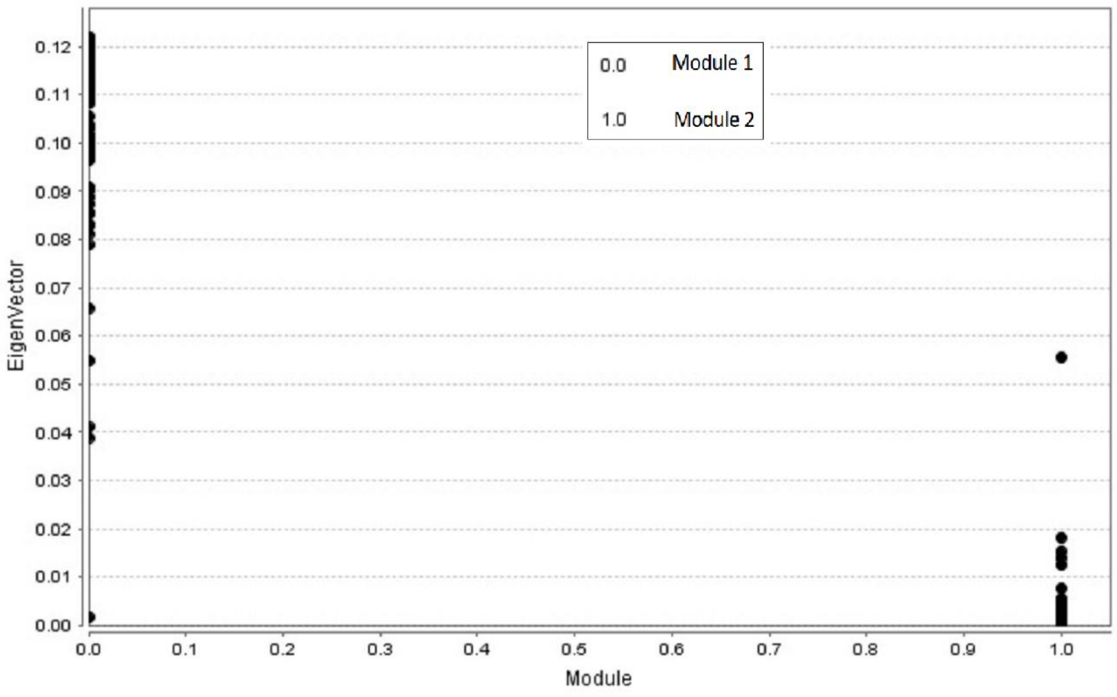

Figure S6. Metrics distribution for each module. (c) Eigenvector distribution.

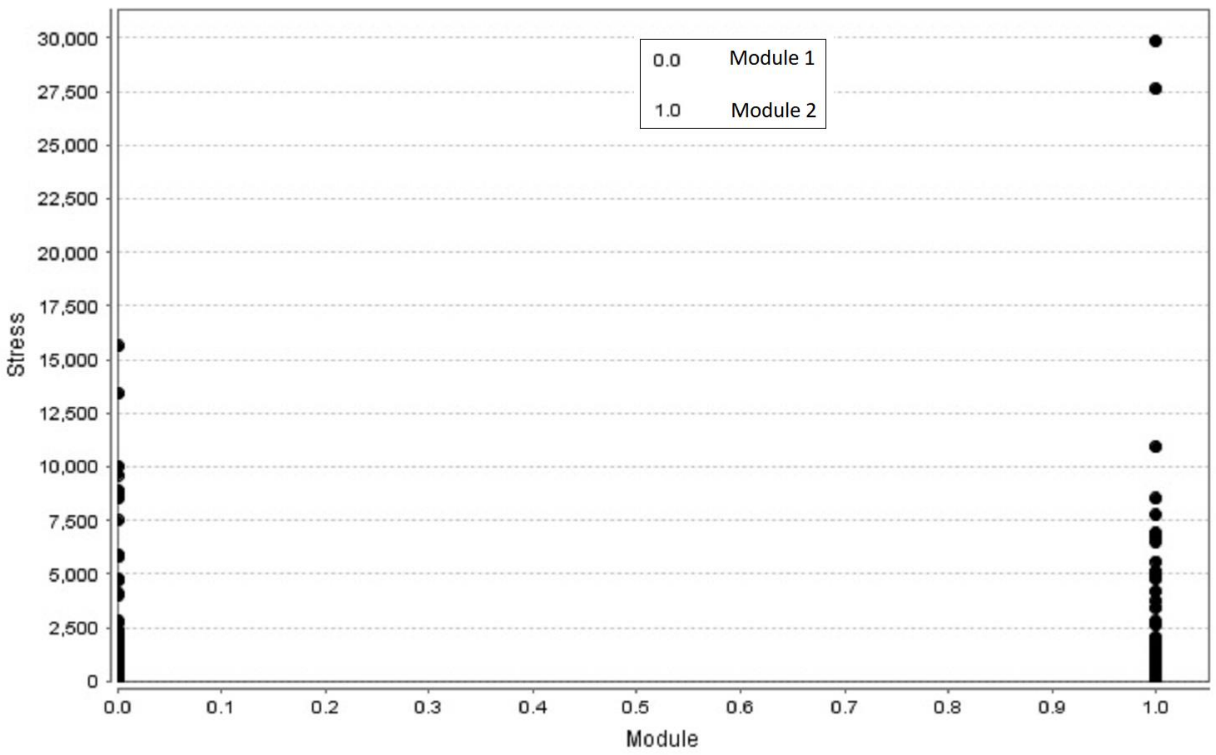

Figure S6. Metrics distribution for each module. (d) Stress distribution.

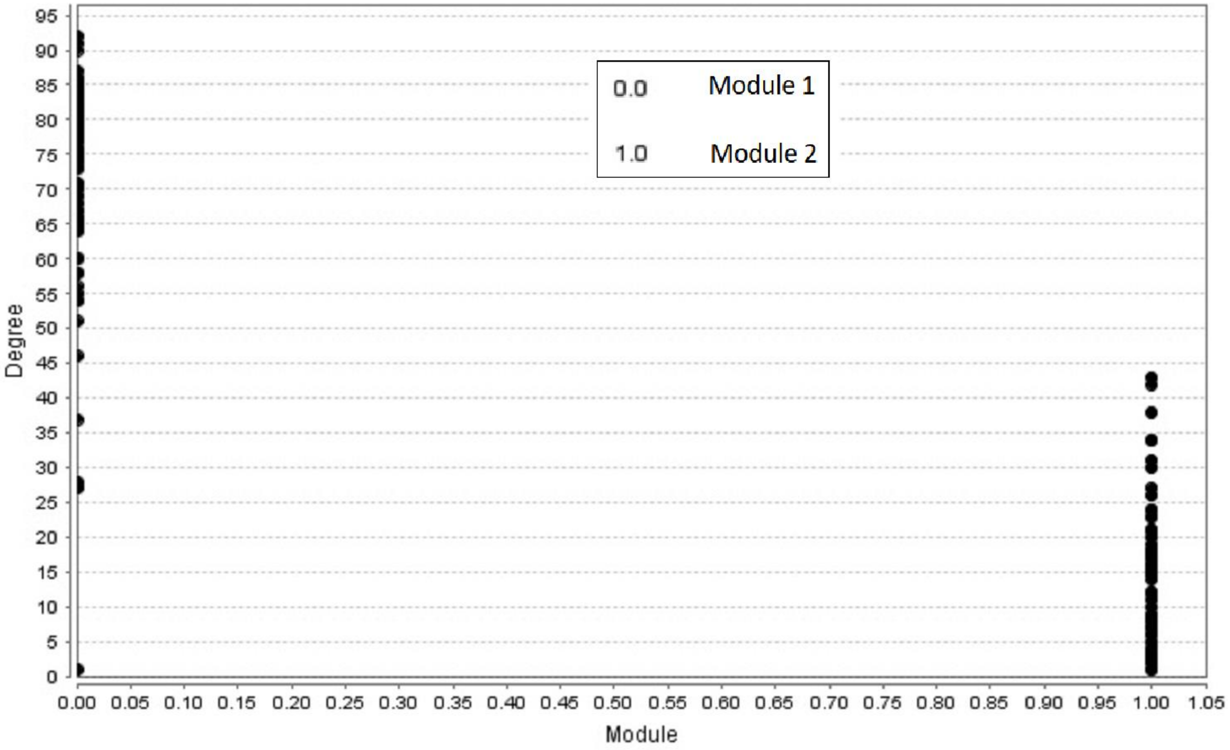

Figure S6. Metrics distribution for each module. (e) Degree distribution.
