# Supplementary material for: Using Micro- and Macro-Level Network Metrics Unveils Top Communicative Gene Modules in Psoriasis
Source: Genes (Basel). 2020 Aug 10;11(8):914. doi: 10.3390/genes11080914 (PMC7464240; doi:10.3390/genes11080914)
Supplement: Supplementary file 1 [file genes-11-00914-s001.zip › supplementary_materials_version2/Table S13.pdf]

Table S13. Intersectional pathways among top 20 enriched pathways based on 149 merged genes from modules 1 and 2 sorted by combined score by WikiPathways 2019 HUMAN Database

**Index**	**Term**	**Genes**	**Combined score**
**1**	IL-4 Signaling Pathway WP395	SOCS3;STAT1;STAT3;BIRC5;PIK3R1;CBL; MAPK14	273.8
**2**	Integrated Cancer Pathway WP1971	BLM;STAT1;CHEK1;PLK1;CDK1;CDC25A	255.5
**3**	Hepatitis C and Hepatocellular Carcinoma WP3646	CXCL8;RRM2;NOS2;STAT3;BIRC5; MAPK14	218.7
**4**	Photodynamic therapy-induced AP-1 survival signaling. WP3611	CCNA2;PDGFRA;IFNG;MAPK14;MCL1; MAPK13	212.4
**5**	Regulation of toll-like receptor signaling pathway WP1449	CXCL10;CXCL11;CXCL9;CXCL8;STAT1; IL1B;PLK1;IRF7;PIK3R1;MAPK14;MAPK13	198.7
