# Supplementary material for: Using Micro- and Macro-Level Network Metrics Unveils Top Communicative Gene Modules in Psoriasis
Source: Genes (Basel). 2020 Aug 10;11(8):914. doi: 10.3390/genes11080914 (PMC7464240; doi:10.3390/genes11080914)
Supplement: Supplementary file 1 [file genes-11-00914-s001.zip › supplementary_materials_version2/Table S11.pdf]

Table S11. Top enriched pathways in module 2 ranked in order of combined score by KEGG 2019 HUMAN Database.

Index	Name	Genes	Combined score
**1**	Influenza A	CXCL8;DDX58;PRKCB;STAT1;PIK3R1;MAPK14;MAPK13;IFIH1;SOCS3;CXCL10;IFNG;OAS1;OAS2;IL1B;OAS3;IRF7	1298.5
**2**	Pertussis	CXCL8;CALML5;NOS2;IL23A;IL1B;IRF1;CALML3;MAPK14;MAPK13	1012.0
**3**	Hepatitis C	DDX58;EGF;STAT1;STAT3;PIK3R1;SOCS3;CXCL10;IFNG;OAS1;OAS2;OAS3;IRF7;PPARA	906.5
**4**	RIG-I-like receptor signaling pathway	IFIH1;CXCL10;CXCL8;DDX58;IRF7;ISG15;MAPK14;MAPK13	860.8
**5**	Toll-like receptor signaling pathway	CXCL10;CXCL9;CXCL11;CXCL8;STAT1;IL1B;IRF7;PIK3R1;MAPK14;MAPK13	845.2
**6**	C-type lectin receptor signaling pathway	CALML5;IL23A;STAT1;IL1B;IRF1;CALML3;PIK3R1;MAPK14;MAPK13	660.1
**7**	Salmonella infection	TJP1;CXCL8;IFNG;NOS2;IL1B;MYH14;MAPK14;MAPK13	650.9
**8**	Prolactin signaling pathway	SOCS3;STAT1;IRF1;STAT3;PIK3R1;MAPK14;MAPK13	632.2
**9**	Prolactin signaling pathway	SOCS3;STAT1;IRF1;STAT3;PIK3R1;MAPK14;MAPK13	632.2
**10**	Epstein-Barr virus infection	LYN;DDX58;STAT1;STAT3;ISG15;PIK3R1;MAPK14;MAPK13;CXCL10;OAS1;OAS2;OAS3;IRF7	629.4
