# Supplementary material for: Using Micro- and Macro-Level Network Metrics Unveils Top Communicative Gene Modules in Psoriasis
Source: Genes (Basel). 2020 Aug 10;11(8):914. doi: 10.3390/genes11080914 (PMC7464240; doi:10.3390/genes11080914)
Supplement: Supplementary file 1 [file genes-11-00914-s001.zip › supplementary_materials_version2/Table S10.pdf]

Table S10. Top 10 enriched pathways in module 2 in order of combined score by Wikipathways 2019 HUMAN database.

Index	Term	Genes	Combined score
**1**	Type II interferon signaling (IFNG) WP619	SOCS3;CXCL10;CXCL9;NOS2;OAS1;IFNG; STAT1;IL1B;IRF1;ISG15	3324.9
**2**	RIG-I-like Receptor Signaling WP3865	IFIH1;CXCL10;CXCL8;IFNG;DDX58;IRF7; ISG15;MAPK14;MAPK13	1388.0
**3**	IL-10 Anti-inflammatory Signaling Pathway WP4495	STAT1;STAT3;HMOX1	948.8
**4**	Toll-like receptor signaling pathway	CXCL10;CXCL11;CXCL9;CXCL8;STAT1;IL1B ;IRF7;PIK3R1;MAPK14;MAPK13	860.1
**5**	Non-genomic actions of 1,25 dihydroxyvitamin D3 WP4341	CXCL8;IFNG;PRKCB;OAS2;STAT1;ISG15; MAPK14;MAPK13	844.5
**6**	Kit receptor signaling pathway WP304	SOCS3;IRS1;LEP;STAT3	796.4
**7**	Leptin signaling pathway WP2034	SOCS3;STAT1;IL1B;LEP;STAT3;PIK3R1; MAPK14;ACACB	770.3
**8**	Prolactin Signaling Pathway WP2037	SOCS3;NOS2;STAT1;IRF1;STAT3;PIK3R1; CBL;MAPK14	770.3
**9**	IL-4 Signaling Pathway WP395	SOCS3;IRS1;STAT1;STAT3;PIK3R1;CBL; MAPK14	629.8
**10**	Interferon type I signaling pathways WP585	SOCS3;STAT1;STAT3;PIK3R1;CBL;MAPK14	629.8
